# Host-Microbiome Interaction in Lung Cancer

**DOI:** 10.3389/fimmu.2021.679829

**Published:** 2021-05-24

**Authors:** Qiang Dong, Eric S. Chen, Chen Zhao, Chengcheng Jin

**Affiliations:** ^1^ Department of Cancer Biology, Perelman School of Medicine, University of Pennsylvania, Philadelphia, PA, United States; ^2^ Thoracic and Gastrointestinal Malignancies Branch, National Cancer Institute, National Institutes of Health, Bethesda, MD, United States

**Keywords:** lung cancer, microbiota, cancer therapy, tumor microenvironment (TME), tumor immunology, Gut-Lung Axis, immunotherapy

## Abstract

Commensal microbiota has emerged as an essential biomarker and regulator of both tumorigenesis and response to cancer therapy. However, our current knowledge about microbiota in cancer has been largely limited to intestinal microbiota. As a mucosal organ harboring one of the largest surface areas in the body, the lung is exposed to a variety of microbes through inhalation and micro-aspiration, and is colonized by a diverse bacterial community in both physiological and pathological conditions. Importantly, increasing evidence has linked the lung microbiome to cancer development. Studies in lung cancer patients and mouse models have revealed tumor-associated dysregulation of the local microbiome in the lung, which in turn impacts cancer progression by shaping the tumor microenvironment and modulating the activity of tumor-infiltrating immune cells. These findings not only provide novel mechanistic insight into the biology of lung cancer but also shed light on new therapeutic targets and strategies for lung cancer prevention and treatment. The goal of this review is to discuss the key findings, remaining questions, and future directions in this new and exciting field.

## Introduction

Mucosal surfaces exposed to the external environment are colonized by a vast number of microbes consisting of bacteria, viruses, fungi and archaea, which are collectively referred to as the commensal microbiota (1, 2). While oral cavity, skin, respiratory tract, urogenital tract and gastrointestinal tract are major body sites that each harbor unique microbiota, the primary habitat of commensal bacteria is the gastrointestinal tract (3). Numerous studies in the past two decades have explored the interactions between the host and microbiome, and have demonstrated the critical role of microbiome in regulating diverse physiological and pathological processes of the host. For example, commensal microbiota was essential in maintaining host metabolic homeostasis, instructing the development of specific organ structures, and driving the maturation of the host immune system (4). On the other hand, dysregulation of the microbiota with altered bacterial composition, quantity, and diversity has been found to increase host susceptibility to various pathogen infections, exacerbate intestinal inflammation and autoimmunity, induce different forms of metabolic disorders, and promote the development of neurological diseases (5, 6). Importantly, the microbiome is increasingly being recognized for its role in multiple aspects of cancer development and treatment across different types of cancer (7).

Lung cancer is the second most common cancer and is the leading cause of cancer-related deaths in both men and women (8). While small cell lung cancer (SCLC) accounts for 10-15% of lung cancer cases, up to 85% of lung cancers are non-small cell lung cancer (NSCLC), of which the main subtypes are adenocarcinoma, squamous cell carcinoma, and large cell carcinoma. At the molecular level, both the genomic landscape and genetic heterogeneity of lung cancer have been extensively characterized, suggesting that it is a highly heterogeneous set of diseases. Despite the recent development of targeted therapies for some genetic subtypes of human lung cancer, overall survival rates for this disease remain very low. Cigarette smoking is the number one risk factor for lung cancer, while other established risk factors may include second-hand smoke, air pollution, exposure to radon, asbestos, and other occupational carcinogens (9, 10). However, the mechanisms by which these environmental risk factors and other tumor-extrinsic factors control lung carcinogenesis remain poorly understood. As the organ with the largest surface area in the human body and with gas exchange functions, the lung is inevitably exposed to various environmental microorganisms. Therefore, the critical questions are: does the lung have its own indigenous microbiota, and do the lung microbiota change with cancer? How do the microbiota impact lung carcinogenesis? What are the underlying mechanisms and any potential clinical implications? Focusing on these key questions, we hereby summarize the recent advances in this rising area of host-microbiome interaction in lung cancer.

## Does the Lung Have Its Own Microbiota?

Lung, as an organ for gas exchange, harbors one of the largest interfaces between the human host and the external environment, and is therefore a unique mucosal site for host-microbiota interaction (11). Yet the healthy lung is traditionally believed to be sterile due to the failure to grow bacteria from lower airway samples using traditional culture-based microbiological approaches. However, it is now recognized that only 20~40% of bacteria in human feces can be cultivated (12). Until very recently, a substantial number of studies applying 16S-based culture-independent analysis have identified the presence of microbial communities containing a complex diversity of bacteria in the lung in both health and diseases (13–18). Firmicutes, Proteobacteria, Bacteroidetes and Actinobacteria are found to be the major phyla of the lung microbiome, while the genera including *Prevotella*, *Veillonella*, *Streptococcus*, *Neisseria*, *Haemophilus, Fusobacterium*, *Sphingomonas*, *Pseudomonas*, *Acinetobacter*, *Megasphaera*, *Staphylococcus*, and *Corynebacterium* have all been described in the airway microbiota (14, 16, 19). About 20% of OTUs observed by sequencing-based technology have been confirmed by culture-based studies (20). In general, the bacterial composition of the lung microbiota is distinct from the gut or skin microbiota, but shares considerable similarity with the upper respiratory tract and oral microbiome (13, 16).

One of the key features of lung microbiota is its low bacterial biomass, especially when compared to the gastrointestinal microbiome (21). Human lungs harbor 2,000 bacterial genomes per cm^2^ of surface area when estimated based on 16S rRNA analysis of bronchial brushings (16). Experimentally, this introduces technical challenges with sequencing, particularly when using techniques designed for high biomass samples, such as gut microbiome analyses. Low biomass can often result in increased erroneous read frequency as well as background 16s rRNA contamination from the environment or reagents used (22). To combat this, certain strategies with different strengths and weaknesses have been developed, such as the exclusion method and correlation analysis. The abundance and composition of the lung microbiome are mainly regulated by the balance of these three factors: bacterial immigration, elimination, and replication (23, 24). Regarding bacterial immigration, there are two major sources of lung microbiota while inhalation constantly exposes the lung to the air-borne bacteria and microbiota from the upper respiratory tract, micro-aspiration of oral fluids also seeds the lungs with oral bacteria. On the other hand, the known elimination pathways of the pulmonary microbes include cough, mucociliary clearance, and innate and adaptive host defenses (23, 24). Lastly, the bacterial replication rate is mostly determined by the regional growth conditions such as oxygen level, pH, nutrient availability, and anatomical location. Importantly, lung diseases can cause changes in the local environment, favoring the growth of certain bacterial species over others, thereby leading to the development of dysbiosis (24).

Emerging evidence has linked local dysbiosis with both acute and chronic pulmonary diseases (25). For example, although the lung microbiome varies across individuals, no spatial variation was observed within the healthy lung (23). In contrast, in patients with advanced chronic obstructive pulmonary disease (COPD), the marked micro-anatomic differences in bacterial communities within the lungs were identified even when there was no change in bronchiectasis by CT scan (14). Similar spatial heterogeneity was also discovered in the lung with cystic fibrosis (26). Besides microbial distribution, both the biomass and composition of the lung microbiome are altered in pathological conditions. In asthmatic patients, the bacterial burden is much higher than the healthy controls, and the relative abundance of specific bacterial families within the community is closely correlated to the severity of bronchial hyperresponsiveness (18). In patients with COPD, the overgrowth of certain pathogenic microorganisms and the appearance of new commensal strains in the lung are associated with disease exacerbation (27, 28).

## Does the Lung Microbiota Play a Role in Lung Cancer?

Multiple lines of clinical evidence have suggested the link between lung microbiota dysbiosis and cancer. Early epidemiology data showed that bacterial infections are very common in lung cancer patients. Up to 50%-70% of lung cancer cases are complicated by pulmonary infections over the course of the disease (29), and post-obstructive pneumonia can negatively affect the efficacy of lung cancer therapy and overall survival of cancer patients (30). However, the microbiology of these pneumonias is generally poorly understood, making antibiotic therapies hard to direct. More recently, with the advent of high throughput sequencing techniques, an increasing number of studies have revealed the strong association between local dysbiosis and lung cancer. For example, in comparison to that of the non-malignant lung tissue samples, the microbiota of lung tumors was found to exhibit substantially lower taxonomic alpha diversity, while the bacterial compositions were correlated with epidemiologic exposures and cancer stages (31). Regarding the specific bacterial taxon, the genus *Thermus* was reported to be more abundant in tumor tissues from advanced-stage patients, and *Legionella* was higher in patients who developed metastases, implicating the role of these bacteria in cancer progression (31). Similarly, various other bacterial taxa have also been associated with lung cancer by a number of studies. Yan et al. showed that the oral microorganisms *Veillonella* and *Capnocytophaga* were significantly higher in the saliva samples of lung cancer patients and that they may be used as biomarkers for early detection of both small-cell carcinoma and adenocarcinoma (32); Lee et al. detected increased abundance of *Veillonella* and *Megasphaera* in bronchoalveolar lavage samples from lung cancer patients (33); Cameron et al. found that *Granulicatella adiacens* and six other opportunistic pathogens were more common in spontaneous sputum samples from lung cancer patients (34); Greathouse and colleagues demonstrated a correlation between *Acidovorax* genus and squamous cell carcinoma, and identified a specific group of taxa enriched in smokers with TP53 mutation (35); Gomes and colleagues described that *Brevundimonas*, *Acinetobacter*, and *Propionibacterium* were more enriched in lung adenocarcinoma while *Enterobacter* was relatively more enriched in squamous-cell carcinoma (19); it was also found that the lower airway samples from lung cancer individuals were enriched more supraglottic-predominant taxa including *Streptococcus*, *Prevotella*, *Veillonella*, and *Rothia* (36).

Overall, although the exact bacterial taxa identified in lung cancer may vary from one study to another depending on the sample type, sampling method, and patient cohort, the consistent findings are that lung cancer is associated with a dysregulated local microbiome, featured by increased total bacterial abundance, reduced alpha-diversity, and altered bacterial composition. While the specific consequences of altered bacterial diversity in lung cancer have yet to be elucidated, previous studies have shown that increased alpha diversity tends to be associated with better survival and treatment response outcomes in certain cancers, such as cervical cancer and resected pancreatic adenocarcinoma, potentially by influencing the host immune response (37, 38). Interestingly, there seems to an overall enrichment of oral bacteria in lung tumors as observed by multiple groups, and certain bacterial taxa are particularly over-represented in specific subtypes of lung cancer and are correlated with smoking status, and genetic mutations. Altogether, these recent studies suggest that the microbiome may soon become a critical diagnostic and preventive biomarker for lung cancer status, stage, host genotypes and risk factors (**Table 1**).

**Table 1:** Lung microbiome and their association with lung cancer.

Bacterial taxa	Types	Sampling method	Detect method	Correlation with Lung Cancer	Potential mechanisms of action in lung cancer
*Pseudomonas* (14, 19, 26, 30, 31)	Gram-negative, Aerobes	Lung explants, BAL, specimen brush, lung tissues	16S rDNA sequencing	Enriched in lung adenocarcinoma (35)	Positively correlated with macrophage abundance and IFN-γ level in the BAL (39), positively correlated with neutrophil elastase activity (40)
*Streptococcus* (13, 14, 16, 23, 26, 34)	Gram-positive, Facultative anaerobes	Lung explants, BAL, lung tissues, specimen brush,	16S rDNA sequencing	Enriched in lung cancer (34), lung adenocarcinoma (19)	Upregulate ERK and PI3K pathway (36), negatively correlated with active neutrophil elastase (40)
*Prevotella* (13, 14, 16, 23, 34)	Gram-negative, Anaerobes	Specimen brush, lung explants, BAL	16S rDNA sequencing	Enriched in lung cancer (34), lung adenocarcinoma (19)	Upregulate ERK and PI3K pathway (36), positively correlated with Th17 cells and neutrophils (39)
*Fusobacterium* (13, 14, 16, 23)	Gram-negative, Anaerobes	Specimen brush, lung explants, BAL	16S rDNA sequencing	N.D.	N.D.
*Veillonella* (13, 14, 26, 32, 33)	Gram-negative, Anaerobes	Specimen brush, lung explants, BAL, lung tissues	16S rDNA sequencing	Enriched in Lung cancer (33), Adenocarcinoma (19) & Squamous cell carcinoma (32)	Upregulate ERK and PI3K pathway (36), positively correlated with Th17 cells and neutrophils (39)
*Porphyromonas* (13, 14, 19)	Gram-negative, Obligate anaerobes	Specimen brush, lung explants, BAL	16S rDNA sequencing	Enriched in Adenocarcinoma (19)	N.D.
*Neisseria* (13, 14, 23)	Gram-negative, Obligate aerobes	Specimen brush, lung explants, BAL	16S rDNA sequencing	Decreased in Lung cancer (32)	Inhibit growth of cancer cells (32)
*Haemophilus* (14, 19)	Gram-negative, Facultative anaerobes	Lung explants, BAL,	16S rDNA sequencing	N.D.	N.D.
*Sphingomonas* (19, 23, 26)	Gram-negative, Strictly aerobes	BAL, specimen brush, lung tissues	16S rDNA sequencing	Enriched in Adenocarcinoma (19)	Positively correlated with macrophage abundance and IFN-γ level in the BAL (39)
*Acinetobacter* (14, 19, 34)	Gram-negative, Strictly aerobes	Lung explants, BAL	16S rDNA sequencing	Enriched in Lung cancer (34), Adenocarcinoma (19)	N.D.
*Staphylococcus* (13, 14, 30)	Gram-positive, Facultative anaerobes	Specimen brush, lung explants, BAL, lung tissues	16S rDNA sequencing	N.D.	N.D.
*Corynebacterium* (13, 14, 19)	Gram-negative, Facultative anaerobes or aerobes	Specimen brush, lung explants, BAL	16S rDNA sequencing	N.D.	N.D.
*Lactobacillus* (19, 26)	Gram-positive, Aerotolerant anaerobes	BAL, specimen brush, lung tissues	16S rDNA sequencing	N.D.	N.D.
*Actinobacillus* (14, 19)	Gram-negative, Facultative anaerobes or aerobes	Lung explants, BAL	16S rDNA sequencing	Enriched in Squamous cell carcinoma (19)	N.D.
*Propionibacterium* (19)	Gram-positive, Facultative anaerobes	BAL	16S rDNA sequencing	N.D.	N.D.
*Ralstonia* (31)	Gram-negative, Aerobes	lung tissues	16S rDNA sequencing	Enriched in Adenocarcinoma (31)	N.D.
*Megasphaera* (33)	Gram-negative, Aerobic or facultative anaerobic	BAL	16S rDNA sequencing	Enriched in Lung cancer (33)	N.D.
*Acidovorax* (35)	Gram-negative, Aerobes	lung tissues	16S rDNA sequencing	Enriched in Squamous cell carcinoma (35)	N.D.

N.D., Not described.

## How Does the Microbiota Impact the Initiation and Progression of Lung Cancer?

**Figure 1:**
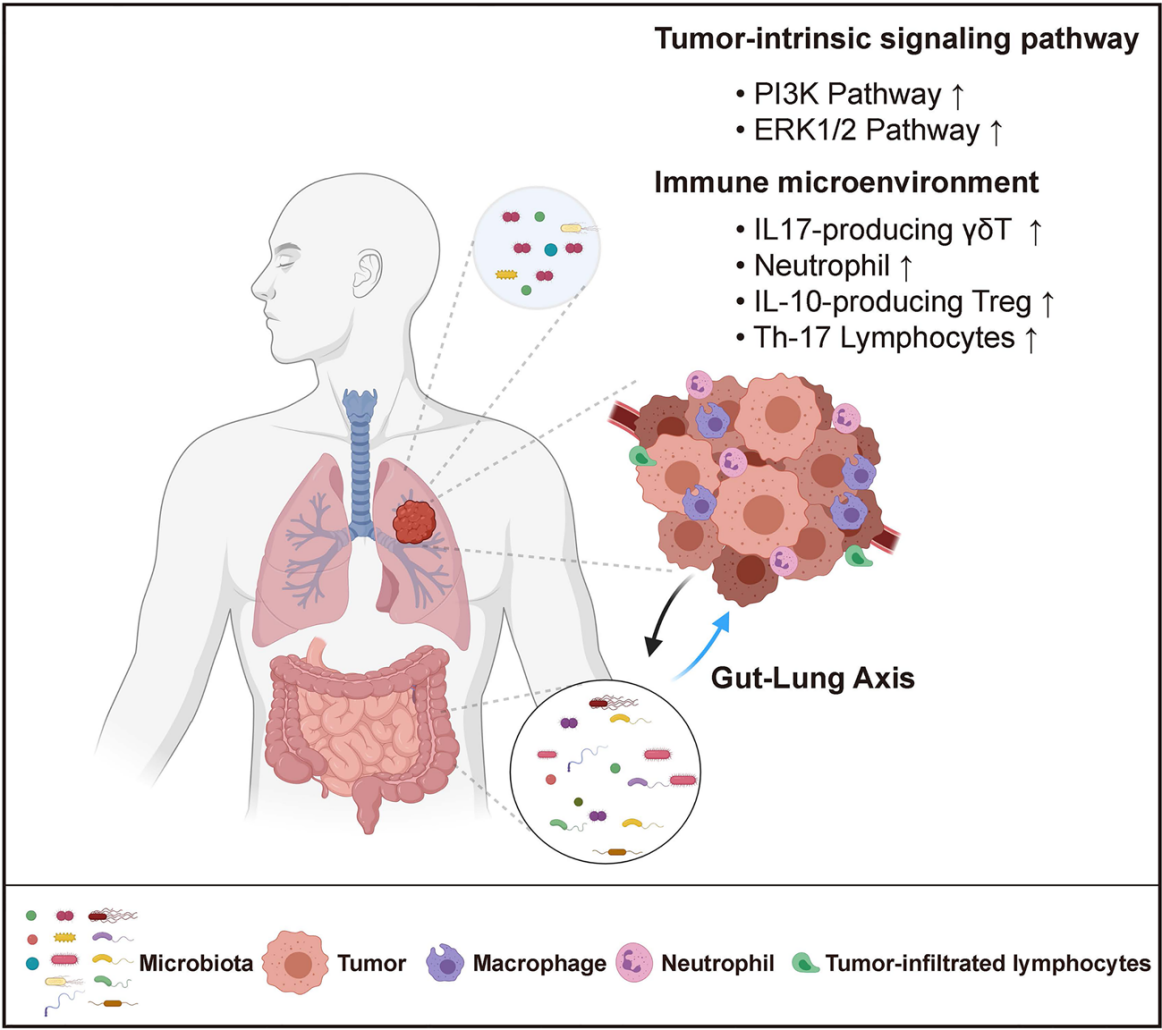
Host-Microbiome Interaction in Lung Cancer. Both local (lung) and distal (gut) microbiota play critical roles in lung cancer. Local microbiota derived metabolites may serve as signaling molecules that directly impact tumor cell intrinsic pathways. They can also reshape the local immune microenvironment by promoting inflammation and inducing immune-suppression. Distal gut microbiota may affect lung tumor development through the regulation of the systemic immune response.

Despite the established link between carcinogenesis and altered lung microbiota, it remains unclear whether the lung microbiome has a causal effect in lung cancer development, and whether it can be a therapeutic target for lung cancer treatment. To address this question, it is key is to elucidate the role of microbiota in lung carcinogenesis and to dissect the mechanisms by which microbiota regulate tumor initiation and progression. Similar to the findings in gut microbiota, it has been suggested that the lung microbiome may contribute to lung tumorigenesis either by directly acting on tumor cells or indirectly *via* modulation of the tumor-associated immune response (**Figure 1**).

Lung microbiota can regulate specific oncogenic pathways that directly drive carcinogenesis. As a number of bacterial metabolites have been implicated in the regulation of host metabolism and signaling pathways (41), it is possible that the alteration of bacteria-derived molecules resulting from dysbiosis in the tumor microenvironment can affect the metabolism and oncogenic signaling of lung cancer cells. Supporting this notion, it was reported that the local microbiota of lung cancer patients exhibited a decreased abundance of the KEGG modules of energy metabolism and ABC-type transport, with increased modules involved in amino acid metabolism, lipid metabolism, xenobiotic biodegradation and metabolism (31, 42). This altered microbial metabolic profile could further impact the gene expressions in airway epithelia cells. In vitro stimulation of the human lung adenocarcinoma cell line A549 with bacterial products isolated from lung cancer patient enriched bacteria was shown to upregulate the expression of genes involved in the PI3K and ERK1/2 signaling pathway, which was consistent with the transcriptomic changes observed in lung cancer patients compared with healthy individuals (36). Importantly, PI3K pathway upregulation has been considered as an early event in the process of lung tumorigenesis (43), suggesting a direct link between lung microbiota and oncogenesis.

Additionally, it has also been demonstrated that the microbiota shape the immune microenvironment in the lung to promote tumor growth. A network of lung-resident immune cells maintains pulmonary tissue homeostasis in the steady-state and provide immune defense in response to invading pathogens (44). The development of lung cancer is closely associated with chronic inflammation characterized by infiltration of inflammatory cells and accumulation of pro-inflammatory factors including cytokines, chemokines and prostaglandins that stimulate cell proliferation, angiogenesis, tissue remodeling, or metastasis (45). However, the source of inflammation has not been clearly defined, and the contribution of specific cellular and molecular components of the immune system remains to be determined. Using an autochthonous GEMM of lung adenocarcinoma driven by an activating point mutation of Kras and loss of p53, our previous study has demonstrated the critical role of microbiota-immune crosstalk in driving inflammation and lung tumorigenesis (46). Mechanistically, we found that lung tumor growth is associated with increased bacterial load and altered bacterial composition in the airway. This dysregulated local microbiota stimulated Myd88-dependent IL-1β and IL-23 production from myeloid cells, inducing proliferation and activation of lung-resident Vγ6+Vδ1+ γδ T cells. These γδ T cells then produced IL-17 to promote neutrophil infiltration and inflammation in the tumor microenvironment; they also expressed IL-22, amphiregulin, and other effector molecules to directly enhance tumor cell proliferation. Eliminating commensal bacteria by systemic antibiotic treatment or rederiving the mice to the germ-free (GF) condition, as well as blocking the γδ T or downstream IL-17 all significantly suppressed tumor growth in the lung. The tumor-promoting role of microbiota elucidated in our study is consistent with the findings from another study showing that treatment of aerosolized vancomycin and neomycin reduced lung tumor implantation. In this study, aerosolized antibiotic treatment led to decreased IL-10 producing Tregs and increased activation of the anti-tumoral NK and T cell response, thereby alleviating the immune suppression in the tumor microenvironment (9).

It is also becoming clear that the specific bacterial composition of the lung is key to regulating lung inflammation, especially in the context of cancer. Interestingly, de-repression of the anti-tumor immunity was found to be associated with a shift from *Firmicutes* to *Proteobacteria* or other Gram-positive microbes rather than specific species after antibiotic treatment (9). Therefore, the balance between different bacterial taxa in the lung seems to be critical for maintaining the local immune homeostasis. Further supporting this, Segal and colleagues found that enrichment of oral bacterial taxa in the lung, such as *Prevotella* and *Veillonella*, was associated with an inflammatory phenotype, including an increased level of Th17 lymphocytes, enhanced expression of inflammatory cytokines, and a blunted alveolar macrophage TLR4 response (39). In addition, a previous study showed that immediately following birth, a shift from a predominance of *Gammaproteobacteria* and *Firmicute*s towards *Bacteroidetes* promoted transient PD-L1 expression in lung CD11b+ CD103- dendritic cells, regulating regulatory T cell activation and the general aeroallergen response in a two-week window after birth (47). Although this specific study did not discuss this effect in cancer, it is likely that shifting bacterial diversity may be able to regulate key molecules like PD-L1 as well as various innate and adaptive immune populations. Altogether, these studies not only provide mechanistic insight into the host-microbiome interaction in lung cancer, but also define novel targets for lung cancer prevention and treatment.

## Does the Gut Microbiota Play a Role in Lung Cancer?

The gastrointestinal tract harbors 99% of the commensal bacterial mass of the human microbiome. The bi-directional crosstalk between the gut and the lung has been well established, and the gut microbiota was previously found to regulate the lung immune response in allergic airway diseases and pulmonary viral infection (48–50). Gut microbiota has also emerged as a biomarker and regulator of both tumorigenesis and host response to cancer therapy. Interestingly, while the gut microbiota is found to promote local inflammation and development of gastrointestinal cancers (7, 51), it seems to exert an opposing effect on transplanted tumors at distal sites by priming the host immune system and enhancing the systemic anti-tumor immune response (52). Using a transplanted mouse model, previous studies showed that oral antibiotic treatment could increase the susceptibility to Lewis lung carcinoma (53), and reduced the anti-cancer response of the chemotherapeutic agent cisplatin (54). In a landmark study by Routy et al., it was shown that the patients with advanced non-small cell lung cancer who took ATB orally shortly before or after the first administration of PD-1/PD-L1 mAb have significantly decreased progression-free survival and overall survival (55). A similar conclusion was also drawn by many other studies (56–58). Given that the intestinal microbiome is the main target of oral antibiotic treatment, these findings suggest that distal intestinal microbiota may indirectly regulate lung cancer development and the therapeutic efficacy of chemotherapy or immunotherapy through regulation of the systemic immune response.

However, these studies mainly focused on load and diversity in the intestinal microbiome and did not specifically study changes in the lung microbiome. Recently, new work has come out specifically describing how lower airway dysbiosis leads to worse NSCLC progression and decreased survival. Tsay et al. showed that *Veillonella parvula* was the most abundant taxon driving this dysbiotic association, leading to upregulation of the IL17, PI3K, MAPK, and ERK pathways in the airway transcriptome. They then used *V. parvula* in the murine KP lung cancer model, characterizing the mechanistic effects on lower airway CD4+ and CD8+, neutrophils, and Th17 recruitment (59). Also, Peters et al. recently demonstrated through 16s rRNA gene sequencing of lung samples from NSCLC patients that lung tumor tissue was correlated with significantly lower bacterial richness and diversity, and that alterations in the relative abundance of families such as *Koribacteraceae*, *Bacteroidaceae*, *Lachnospiraceae*, and *Ruminococcaceae* were associated with increased or decreased recurrence-free and disease-free survival (60). It is clear that particularly in lung cancer patients, it may be necessary to conduct deeper analysis specifically into the role of lung bacteria and seek to isolate these effects from the gut.

On the other hand, lung cancer development is also associated with changes in the gut microbiome. A recent study found a significant difference in the beta-diversity but not the alpha diversity of the gut microbiota between lung cancer patients and healthy control individuals, with a particular over-representation of *Enterococcus* in the fecal material from lung cancer patients (61). Similarly, several other bacteria tax, including *Streptococcus*, *Prevotella*, *Blautia, Coprococcus, Bifidobacterium*, and *Lachnospiraceae* were found to be enriched in the gut microbiota of lung cancer patients (42). Additionally, Ming et al. showed that the gut microbiome in lung cancer patients have a lower abundance of *Prevotella* but a higher level of *Actinomyces* and *Streptococcus*. Interestingly, the alteration of the gut microbiome in lung cancer patients seems to be dependent on the different lung cancer subtypes: lower alpha diversity of the gut microbiome was only observed in patients with small cell lung cancer but not adenocarcinoma or squamous cell carcinoma; in terms of the taxonomical composition, gut microbiota in adenocarcinoma patients was characterized by the dominant phylotypes including *Bifidobacterium*, *Clostridium*, and *Prevotella*, squamous cell carcinoma patients exhibited a high abundance of *Ruminococcus*, *Lachnospira*, and *Lactobacillus* in their gut, and *Streptococcus*, *Anaerotruncus*, and *Bacillus* were enriched in small cell lung cancer (62). Taken together, these observations indicate that the distal gut microbiome may also serve as a robust biomarker for lung cancer.

## Future Directions

Based on our current knowledge about the host-microbiome interaction in lung cancer, the research in this area is progressing towards these two main directions: first is to understand how the microbiota co-evolves with tumors during cancer initiation and progression to identify the changes in bacterial composition, quantity, diversity and metabolic activity that can be used as reliable biomarkers for lung cancer diagnosis; second is to further elucidate the cellular and molecular mediators of host-microbiota interaction that can be therapeutically targeted for lung cancer treatment.

Supporting the idea that specific bacterial species may serve as unique biomarkers for lung cancer subtypes and tumor stages, Greathouse et al. showed the higher abundance of *Acidovorax* among the subset of squamous cell carcinoma cases with TP53 mutations, but this association was not present in adenocarcinomas. Another study analyzed the lower airway microbiota in patients undergoing diagnostic bronchoscopy for suspected lung cancer and found that malignancy could be determined with an accuracy of 70% by isolating *Enterococcus, Capnocytophaga*, or *Actinomyces*, whereas benign diseases can be indicated by Microbispora with a sensitivity of 55%, specificity of 88%, and accuracy of 78% (63). Similarly, enrichment of *Capnocytophaga* and *Veillonella* were observed in both small cell lung cancer and lung adenocarcinoma, and the combination of these two bacterial biomarkers succeeded in distinguishing patients with small cell lung cancer or lung adenocarcinoma from healthy individuals (32).

It is worth mentioning that in one of the latest studies, Nejman and colleagues performed a comprehensive analysis of the tumor microbiome across seven cancer types, including lung cancer (64). Importantly, bacterial components were detected within lung tumors as measured by 16S qPCR, IHC for LPS, and *in situ* hybridization of 16S rRNA. While each tumor type was shown to have distinct microbial compositions, it was found that bacterial taxa that can degrade chemicals in cigarette smoke were significantly enriched in lung tumors compared with other tumor types, suggesting that high levels of these metabolites create a preferred niche for bacteria that can use these molecules. This enrichment was even more apparent in lung tumors from current smokers in comparison to never-smokers. Moreover, bacterial pathways involved in the biosynthesis of plant metabolites present in cigarette tobacco were also enriched in the lung tumors of smokers, further reflecting the impact of environmental exposure on the lung microbiome. Specifically, with regards to smoking, many different studies have observed significant changes to the lung microbiome in different smoking condition. While some have specifically shown a positive correlation between smoking and alpha diversity, others have shown specific enhancement or reduction in key populations such as *Pseudomonas, Streptococcus*, and *Granulicatella* (14, 31, 65). This suggests that in addition to the direct effects of smoking and other environmental factors on tumor cells, the smoking-microbiome-immune axis also likely plays an important role in regulating overall tumor progression.

Moving forward, we think the critical questions in this field that require further investigations are (1) How do tumors shape the local microbial community in the lung? (2) What are the specific tumor-promoting component(s) in lung microbiota? To this end, it is critical to interrogate the interactions between the microbiome and host genes, as well as the interactions between the microbiome and environmental exposure such as cigarette smoking. Furthermore, given the potent impact of gut microbiome on the efficacy of various anti-cancer therapies including the lung cancer response to cisplatin and immune checkpoint blockade, it is also essential to further delineate the specific roles of the local lung microbiome *vs*. the distal gut microbiome in tumor growth and tumor-associated immune responses. It is possible that selectively targeting these two compartments may exert distinct effects on lung cancer progression and treatment, thereby shedding light on new strategies for future lung cancer therapies.

## Author Contributions

QD, ESC, CZ, and CJ wrote the manuscript. All authors contributed to the article and approved the submitted version. 

## Funding

CJ is supported in part by a R00 award from NIH/NCI (CA226400), an Emerson Collective Cancer Research fund, and a Lung Cancer Research Foundation (LCRF) pilot grant. CZ is supported in part by the Intramural Research Program of the NIH, National Cancer Institute, Center for Cancer Research, SITC-AstraZeneca Immunotherapy in Lung Cancer (Early Stage NSCLC) Clinical Fellowship Award. CJ and CZ are supported by NIH Bench-to-Bedside and Back Program (BtB).

## Conflict of Interest

The authors declare that the research was conducted in the absence of any commercial or financial relationships that could be construed as a potential conflict of interest.

## References

[1] Gilbert JA Blaser MJ Caporaso JG Jansson JK Lynch SV Knight R. Current Understanding of the Human Microbiome. Nat Med (2018) 24(4):392–400. 10.1038/nm.4517 29634682 PMC7043356

[2] Ursell LK Metcalf JL Parfrey LW Knight R. Defining the Human Microbiome. Nutr Rev (2012) 70(Suppl 1):S38–44. 10.1111/j.1753-4887.2012.00493.x PMC3426293 22861806

[3] Human Microbiome Project C. Structure, Function and Diversity of the Healthy Human Microbiome. Nature (2012) 486(7402):207–14. 10.1038/nature11234 PMC3564958 22699609

[4] Belkaid Y Hand TW. Role of the Microbiota in Immunity and Inflammation. Cell (2014) 157(1):121–41. 10.1016/j.cell.2014.03.011 PMC4056765 24679531

[5] Butler MI Cryan JF Dinan TG. Man and the Microbiome: A New Theory of Everything? Annu Rev Clin Psychol (2019) 15:371–98. 10.1146/annurev-clinpsy-050718-095432 30786244

[6] Wang B Yao M Lv L Ling Z Li L. The Human Microbiota in Health and Disease. Engineering (2017) 3(1):71–82. 10.1016/j.Eng.2017.01.008

[7] Garrett WS. Cancer and the Microbiota. Science (2015) 348(6230):80–6. 10.1126/science.aaa4972 PMC5535753 25838377

[8] Siegel RL Miller KD Jemal A. Cancer Statistics, 2019. CA Cancer J Clin (2019) 69(1):7–34. 10.3322/caac.21551 30620402

[9] Le Noci V Guglielmetti S Arioli S Camisaschi C Bianchi F Sommariva M. Modulation of Pulmonary Microbiota by Antibiotic or Probiotic Aerosol Therapy: A Strategy to Promote Immunosurveillance Against Lung Metastases. Cell Rep (2018) 24(13):3528–38. 10.1016/j.celrep.2018.08.090 30257213

[10] Kaderbhai C Richard C Fumet JD Aarnink A Foucher P Coudert B. Antibiotic Use Does Not Appear to Influence Response to Nivolumab. Anticancer Res (2017) 37(6):3195–200. 10.21873/anticanres.11680 28551664

[11] Pilette C Ouadrhiri Y Godding V Vaerman JP Sibille Y. Lung Mucosal Immunity: Immunoglobulin-a Revisited. Eur Respir J (2001) 18(3):571–88. 10.1183/09031936.01.00228801 11589357

[12] Suau A Bonnet R Sutren M Godon JJ Gibson GR Collins MD. Direct Analysis of Genes Encoding 16S rRNA From Complex Communities Reveals Many Novel Molecular Species Within the Human Gut. Appl Environ Microbiol (1999) 65(11):4799–807. 10.1128/AEM.65.11.4799-4807.1999 PMC91647 10543789

[13] Charlson ES Bittinger K Haas AR Fitzgerald AS Frank I Yadav A. Topographical Continuity of Bacterial Populations in the Healthy Human Respiratory Tract. Am J Respir Crit Care Med (2011) 184(8):957–63. 10.1164/rccm.201104-0655OC PMC3208663 21680950

[14] Erb-Downward JR Thompson DL Han MK Freeman CM McCloskey L Schmidt LA. Analysis of the Lung Microbiome in the “Healthy” Smoker and in COPD. PloS One (2011) 6(2):e16384. 10.1371/journal.pone.0016384 21364979 PMC3043049

[15] Harris JK De Groote MA Sagel SD Zemanick ET Kapsner R Penvari C. Molecular Identification of Bacteria in Bronchoalveolar Lavage Fluid From Children With Cystic Fibrosis. Proc Natl Acad Sci U S A (2007) 104(51):20529–33. 10.1073/pnas.0709804104 PMC2154465 18077362

[16] Hilty M Burke C Pedro H Cardenas P Bush A Bossley C. Disordered Microbial Communities in Asthmatic Airways. PloS One (2010) 5(1):e8578. 10.1371/journal.pone.0008578 20052417 PMC2798952

[17] Huang YJ Kim E Cox MJ Brodie EL Brown R Wiener-Kronish JP. A Persistent and Diverse Airway Microbiota Present During Chronic Obstructive Pulmonary Disease Exacerbations. OMICS (2010) 14(1):9–59. 10.1089/omi.2009.0100 20141328 PMC3116451

[18] Huang YJ Nelson CE Brodie EL Desantis TZ Baek MS Liu J. Airway Microbiota and Bronchial Hyperresponsiveness in Patients With Suboptimally Controlled Asthma. J Allergy Clin Immunol (2011) 127(2):372–81.e1-3. 10.1016/j.jaci.2010.10.048 21194740 PMC3037020

[19] Gomes S Cavadas B Ferreira JC Marques PI Monteiro C Sucena M. Profiling of Lung Microbiota Discloses Differences in Adenocarcinoma and Squamous Cell Carcinoma. Sci Rep (2019) 9(1):12838. 10.1038/s41598-019-49195-w 31492894 PMC6731246

[20] Venkataraman A Bassis CM Beck JM Young VB Curtis JL Huffnagle GB. Application of a Neutral Community Model to Assess Structuring of the Human Lung Microbiome. mBio (2015) 6(1):e02284-14. 10.1128/mBio.02284-14 25604788 PMC4324308

[21] Mathieu E Escribano-Vazquez U Descamps D Cherbuy C Langella P Riffault S. Paradigms of Lung Microbiota Functions in Health and Disease, Particularly, in Asthma. Front Physiol (2018) 9:1168. 10.3389/fphys.2018.01168 30246806 PMC6110890

[22] Claassen-Weitz S Gardner-Lubbe S Mwaikono KS du Toit E Zar HJ Nicol MP. Optimizing 16S rRNA Gene Profile Analysis From Low Biomass Nasopharyngeal and Induced Sputum Specimens. BMC Microbiol (2020) 20(1):113. 10.1186/s12866-020-01795-7 32397992 PMC7218582

[23] Dickson RP Erb-Downward JR Freeman CM McCloskey L Beck JM Huffnagle GB. Spatial Variation in the Healthy Human Lung Microbiome and the Adapted Island Model of Lung Biogeography. Ann Am Thorac Soc (2015) 12(6):821–30. 10.1513/AnnalsATS.201501-029OC PMC4590020 25803243

[24] Dickson RP Martinez FJ Huffnagle GB. The Role of the Microbiome in Exacerbations of Chronic Lung Diseases. Lancet (2014) 384(9944):691–702. 10.1016/S0140-6736(14)61136-3 25152271 PMC4166502

[25] O’Dwyer DN Dickson RP Moore BB. The Lung Microbiome, Immunity, and the Pathogenesis of Chronic Lung Disease. J Immunol (2016) 196(12):4839–47. 10.4049/jimmunol.1600279 PMC4894335 27260767

[26] Willner D Haynes MR Furlan M Schmieder R Lim YW Rainey PB. Spatial Distribution of Microbial Communities in the Cystic Fibrosis Lung. ISME J (2012) 6(2):471–4. 10.1038/ismej.2011.104 PMC3260497 21796216

[27] Rosell A Monso E Soler N Torres F Angrill J Riise G. Microbiologic Determinants of Exacerbation in Chronic Obstructive Pulmonary Disease. Arch Intern Med (2005) 165(8):891–7. 10.1001/archinte.165.8.891 15851640

[28] Sethi S Sethi R Eschberger K Lobbins P Cai X Grant BJ. Airway Bacterial Concentrations and Exacerbations of Chronic Obstructive Pulmonary Disease. Am J Respir Crit Care Med (2007) 176(4):356–61. 10.1164/rccm.200703-417OC 17478618

[29] Akinosoglou KS Karkoulias K Marangos M. Infectious Complications in Patients With Lung Cancer. Eur Rev Med Pharmacol Sci (2013) 17(1):8–18. 23329518

[30] Qiao D Wang Z Lu Y Wen X Li H Zhao H. A Retrospective Study of Risk and Prognostic Factors in Relation to Lower Respiratory Tract Infection in Elderly Lung Cancer Patients. Am J Cancer Res (2015) 5(1):423–32. PMC4300720 25628950

[31] Yu G Gail MH Consonni D Carugno M Humphrys M Pesatori AC. Characterizing Human Lung Tissue Microbiota and its Relationship to Epidemiological and Clinical Features. Genome Biol (2016) 17(1):163. 10.1186/s13059-016-1021-1 27468850 PMC4964003

[32] Yan X Yang M Liu J Gao R Hu J Li J. Discovery and Validation of Potential Bacterial Biomarkers for Lung Cancer. Am J Cancer Res (2015) 5(10):3111–22. PMC4656734 26693063

[33] Lee SH Sung JY Yong D Chun J Kim SY Song JH. Characterization of Microbiome in Bronchoalveolar Lavage Fluid of Patients With Lung Cancer Comparing With Benign Mass Like Lesions. Lung Cancer (2016) 102:89–95. 10.1016/j.lungcan.2016.10.016 27987594

[34] Cameron SJS Lewis KE Huws SA Hegarty MJ Lewis PD Pachebat JA. A Pilot Study Using Metagenomic Sequencing of the Sputum Microbiome Suggests Potential Bacterial Biomarkers for Lung Cancer. PloS One (2017) 12(5):e0177062. 10.1371/journal.pone.0177062 28542458 PMC5444587

[35] Greathouse KL White JR Vargas AJ Bliskovsky VV Beck JA von Muhlinen N. Interaction Between the Microbiome and TP53 in Human Lung Cancer. Genome Biol (2018) 19(1):123. 10.1186/s13059-018-1501-6 30143034 PMC6109311

[36] Tsay JJ Wu BG Badri MH Clemente JC Shen N Meyn P. Airway Microbiota is Associated With Upregulation of the PI3K Pathway in Lung Cancer. Am J Respir Crit Care Med (2018) 198(9):1188–98. 10.1164/rccm.201710-2118OC PMC6221574 29864375

[37] Sims TT El Alam MB Karpinets TV Dorta-Estremera S Hegde VL Nookala S. Gut Microbiome Diversity is an Independent Predictor of Survival in Cervical Cancer Patients Receiving Chemoradiation. Commun Biol (2021) 4(1):237. 10.1038/s42003-021-01741-x 33619320 PMC7900251

[38] Riquelme E Zhang Y Zhang L Montiel M Zoltan M Dong W. Tumor Microbiome Diversity and Composition Influence Pancreatic Cancer Outcomes. Cell (2019) 178(4):795–806 e12. 10.1016/j.cell.2019.07.008 31398337 PMC7288240

[39] Segal LN Clemente JC Tsay JC Koralov SB Keller BC Wu BG. Enrichment of the Lung Microbiome With Oral Taxa is Associated With Lung Inflammation of a Th17 Phenotype. Nat Microbiol (2016) 1:16031. 10.1038/nmicrobiol.2016.31 27572644 PMC5010013

[40] Oriano M Gramegna A Terranova L Sotgiu G Sulaiman I Ruggiero L. Sputum Neutrophil Elastase Associates With Microbiota and P. Aeruginosa in Bronchiectasis. Eur Respir J (2020) 56:2000769. 10.1183/13993003.00769-2020 32499333

[41] Nicholson JK Holmes E Kinross J Burcelin R Gibson G Jia W. Host-Gut Microbiota Metabolic Interactions. Science (2012) 336(6086):1262–7. 10.1126/science.1223813 22674330

[42] Liu F Li J Guan Y Lou Y Chen H Xu M. Dysbiosis of the Gut Microbiome is Associated With Tumor Biomarkers in Lung Cancer. Int J Biol Sci (2019) 15(11):2381–92. 10.7150/ijbs.35980 PMC6775324 31595156

[43] Gustafson AM Soldi R Anderlind C Scholand MB Qian J Zhang X. Airway PI3K Pathway Activation is an Early and Reversible Event in Lung Cancer Development. Sci Trans Med (2010) 2(26):26ra5–ra5. 10.1126/scitranslmed.3000251 PMC3694402 20375364

[44] Lloyd CM Marsland BJ. Lung Homeostasis: Influence of Age, Microbes, and the Immune System. Immunity (2017) 46(4):549–61. 10.1016/j.immuni.2017.04.005 28423336

[45] Palucka AK Coussens LM. The Basis of Oncoimmunology. Cell (2016) 164(6):1233–47. 10.1016/j.cell.2016.01.049 PMC4788788 26967289

[46] Jin C Lagoudas GK Zhao C Bullman S Bhutkar A Hu B. Commensal Microbiota Promote Lung Cancer Development Via Gammadelta T Cells. Cell (2019) 176(5):998–1013.e16. 10.1016/j.cell.2018.12.040 30712876 PMC6691977

[47] Gollwitzer ES Saglani S Trompette A Yadava K Sherburn R McCoy KD. Lung Microbiota Promotes Tolerance to Allergens in Neonates Via PD-L1. Nat Med (2014) 20(6):642–7. 10.1038/nm.3568 24813249

[48] Anand S Mande SS. Diet, Microbiota and Gut-Lung Connection. Front Microbiol (2018) 9:2147. 10.3389/fmicb.2018.02147 30283410 PMC6156521

[49] Dumas A Bernard L Poquet Y Lugo-Villarino G Neyrolles O. The Role of the Lung Microbiota and the Gut-Lung Axis in Respiratory Infectious Diseases. Cell Microbiol (2018) 20(12):e12966. 10.1111/cmi.12966 30329198

[50] Vaughan A Frazer ZA Hansbro PM Yang IA. COPD and the Gut-Lung Axis: The Therapeutic Potential of Fibre. J Thorac Dis (2019) 11(Suppl 17):S2173–80. 10.21037/jtd.2019.10.40 PMC6831926 31737344

[51] Elinav E Nowarski R Thaiss CA Hu B Jin C Flavell RA. Inflammation-Induced Cancer: Crosstalk Between Tumours, Immune Cells and Microorganisms. Nat Rev Cancer (2013) 13(11):759–71. 10.1038/nrc3611 24154716

[52] Dzutsev A Badger JH Perez-Chanona E Roy S Salcedo R Smith CK. Microbes and Cancer. Annu Rev Immunol (2017) 35:199–228. 10.1146/annurev-immunol-051116-052133 28142322

[53] Cheng M Qian L Shen G Bian G Xu T Xu W. Microbiota Modulate Tumoral Immune Surveillance in Lung Through a gammadeltaT17 Immune Cell-Dependent Mechanism. Cancer Res (2014) 74(15):4030–41. 10.1158/0008-5472.CAN-13-2462 24947042

[54] Gui QF Lu HF Zhang CX Xu ZR Yang YH. Well-Balanced Commensal Microbiota Contributes to Anti-Cancer Response in a Lung Cancer Mouse Model. Genet Mol Res (2015) 14(2):5642–51. 10.4238/2015.May.25.16 26125762

[55] Routy B Le Chatelier E Derosa L Duong CPM Alou MT Daillere R. Gut Microbiome Influences Efficacy of PD-1-based Immunotherapy Against Epithelial Tumors. Science (2018) 359(6371):91–7. 10.1126/science.aan3706 29097494

[56] Ahmed J Kumar A Parikh K Anwar A Knoll BM Puccio C. Use of Broad-Spectrum Antibiotics Impacts Outcome in Patients Treated With Immune Checkpoint Inhibitors. Oncoimmunology (2018) 7(11):e1507670. 10.1080/2162402X.2018.1507670 30377571 PMC6205076

[57] Derosa L Hellmann MD Spaziano M Halpenny D Fidelle M Rizvi H. Negative Association of Antibiotics on Clinical Activity of Immune Checkpoint Inhibitors in Patients With Advanced Renal Cell and non-Small-Cell Lung Cancer. Ann Oncol (2018) 29(6):1437–44. 10.1093/annonc/mdy103 PMC6354674 29617710

[58] Zhao S Gao G Li W Li X Zhao C Jiang T. Antibiotics are Associated With Attenuated Efficacy of anti-PD-1/PD-L1 Therapies in Chinese Patients With Advanced non-Small Cell Lung Cancer. Lung Cancer (2019) 130:10–7. 10.1016/j.lungcan.2019.01.017 30885328

[59] Tsay JJ Wu BG Sulaiman I Gershner K Schluger R Li Y. Lower Airway Dysbiosis Affects Lung Cancer Progression. Cancer Discov (2021) 11(2):293–307. 10.1158/2159-8290.CD-20-0263 33177060 PMC7858243

[60] Peters BA Hayes RB Goparaju C Reid C Pass HI Ahn J. The Microbiome in Lung Cancer Tissue and Recurrence-Free Survival. Cancer Epidemiol Biomarkers Prev (2019) 28(4):731–40. 10.1158/1055-9965.EPI-18-0966 PMC6449216 30733306

[61] Zhuang H Cheng L Wang Y Zhang YK Zhao MF Liang GD. Dysbiosis of the Gut Microbiome in Lung Cancer. Front Cell Infect Microbiol (2019) 9:112. 10.3389/fcimb.2019.00112 31065547 PMC6489541

[62] Ming L Fang Y Xiaohui C Huan Z Xiaoqing W Yinhui L. Alteration of Gut Microbiome in Lung Cancer Patients. bioRxiv (2019). 10.1101/640359

[63] Sanchez-Hellin V Galiana A Zamora-Molina L Soler-Sempere M Grau-Delgado J Barbera VM. Lower Airway Microbiota and Lung Cancer. Microbiol Biotechnol Lett (2019) 47(3):441–8. 10.4014/mbl.1811.11002

[64] Nejman D Livyatan I Fuks G Gavert N Zwang Y Geller LT. The Human Tumor Microbiome is Composed of Tumor Type-Specific Intracellular Bacteria. Science (2020) 368(6494):973–80. 10.1126/science.aay9189 PMC7757858 32467386

[65] Hosgood HD, 3rd Sapkota AR Rothman N Rohan T Hu W Xu J. The Potential Role of Lung Microbiota in Lung Cancer Attributed to Household Coal Burning Exposures. Environ Mol Mutagen (2014) 55(8):643–51. 10.1002/em.21878 PMC4217127 24895247

